# National survey of the use of cardiac output monitoring tool in general adult ICUs in the United Kingdom

**DOI:** 10.1186/cc9452

**Published:** 2011-03-11

**Authors:** O Couppis, S Saha, E Makings

**Affiliations:** 1 Broomfield Hospital, Chelmsford, UK

## Introduction

Haemodynamic monitoring is essential for the management of critically ill patients. Currently there are various techniques available in clinical practice to measure cardiac output (CO) in ICUs including pulmonary artery catheter (PAC), oesophageal Doppler, lithium dilution cardiac output (LiDCO) and pulse-induced contour cardiac output (PiCCO) studies. In recent times PAC has been used less with less invasive methods becoming more popular. We conducted a telephone survey of the current CO monitoring practices in adult ICUs in the United Kingdom.

## Methods

All general adult ICUs in the United Kingdom were surveyed via telephone. The nurse-in-charge or the senior physician for the shift was consulted to ascertain which cardiac output monitors (COMs) were available for use, which was their first choice and if they used PAC in the past 12 months.

## Results

A total of 225 adult ICUs were surveyed and all the replies were recorded on paper (98% response). Two hundred and eleven (96%) units used at least one form of COM while the rest of the 14 units did not use any COM tool. One hundred and two (48%) use more than one form of cardiac output monitoring. Oesphageal Doppler was most popular (86/211, 41%), followed by LiDCO and PICCO both used in 73/211 (35%) of the units, and pulse contour analysis (14/109, 7%). Seven out of 211 (3%) units still use PAC as the preferred method of COM, of these two had other COM devices available and five used PAC only. Forty-six out of 211 (22%) units were using PAC at least occasionally. In contrast, a similar survey performed in 2005 [1] found PAC (76%) and oesophageal Doppler (53%) devices to be most commonly available. Among the other techniques. 33% of the ICUs use PiCCO and a further 19% use LiDCO systems for CO monitoring (Table 1).

**Table 1:** Frequency of cardiac output monitoring across the United Kingdom

	**2005 **[1]	2010
PAC	76%	22% (46)
Doppler	53%	41% (86)
LiDCO	19%	35% (73)
PICCO	n/a	35% (73)
WC analysis	33%	7% (14)
Other	8%	n/a

## Conclusions

The results show the changes in COM over the past 5 years in comparison with a previous survey in 2005 [1]. There appears to be a steady decline in the use of PACs, with oesophageal Doppler becoming the most popular method of COM. LiDCO and PiCCO are used equally throughout the United Kingdom, with pulse contour analysis becoming less popular.
